# Editorial: Neurocognitive deficits in psychiatric disorders

**DOI:** 10.3389/fpsyt.2023.1242000

**Published:** 2023-08-10

**Authors:** Jie Yang

**Affiliations:** Department of Psychiatry, National Clinical Research Center for Mental Disorders, and National Center for Mental Disorders, The Second Xiangya Hospital of Central South University, Changsha, Hunan, China

**Keywords:** attention bias modification (ABM), GABAergic interneurons, transcranial direct current stimulation (TDCS), thalamus, conscious emotion processing

Neurocognitive impairments are prevalent across various psychiatric disorders, such as schizophrenia, bipolar disorder, depression, and etc. It has been observed during acute illness episodes and can persist in a subset of patients even after remission from psychopathological symptoms. Despite receiving treatment, individuals with psychiatric disorders often experience persistent residual neurocognitive deficits, which significantly impact their quality of life and may contribute to recurring relapses. Research indicates that these neurocognitive impairments can serve as predictors of poor treatment response. Proper assessment and management of neurocognitive deficits in psychiatric disorders is essential to optimize the treatment of these deficits. The research in these fields may deepen our understanding of the neurobiology and neuropsychology mechanism of cognitive deficits in psychiatric disorders, and these findings may have significant implications for promoting the development of cognitive interventions.

In this editorial, we aim to organize and summarize the research literature published in this Special Research Topic**—***Neurocognitive Deficits in Psychiatric Disorders*. Research literature published within this topic are ranged from looking into the neurobiology mechanism of the neurocognition, meta-analysis of the effects of attentional bias modification, animal models, and brain stimulation. It should be noted some research literature published in this Research Topic has expand to investigate the neurocognitive deficits into neurological disorders.

Zeng et al. have used the thalamic subregions that defined by the Brainnetome altas as seed to conduct the whole-brain functional connectivity analysis, and then investigated the shared and distinct abnormities in thalamic-cortical circuits between bipolar depression and remission. The thalamus plays a critical role in emotion regulation and cognition processing, servers as the center of the bottom-up transmission and top-down regulation of the brain. They reported that bipolar patients during depressive state specifically exhibited decreased connectivity in the prefronto-thalamic circuit, which is widely considered as a cognitive-related circuit and its disruption may impair the emotion regulation and cognition reactions. This finding also implying that the disrupted functional connectivity in prefronto-thalamic circuit is a state-related characteristic of bipolar disorder.

Gou et al. have shed an insight into the underlying neurophysiological mechanism of conscious processing of emotion in depressive disorder by adopting activation likelihood estimation analysis. They observed that a wide range of brain areas, including the middle temporal gyrus, superior temporal gyrus, parahippocampal gyrus, superior temporal gyrus, and inferior parietal lobule, were associated with deficits in conscious emotional processing of depressive patients. Meanwhile, these brain regions respond differently to positive or negative stimuli.

Xia et al. conducted meta-analysis to investigate the efficacy of attention bias modification in depression, and explore the optimal protocol of attention bias modification (ABM). They reported that ABM had a greater effect in improving depression and rumination, but no significant differences were observed between ABM and attention control training in terms of attentional control outcome.

Chen et al. have examined the effect of muscarinic acetylcholine receptors (mAChRs) on the excitatory synaptic transmissions of two major subtypes of GABAergic interneurons [i.e., the somatostatin (SST) and parvalbumin (PV) expressing interneurons] in the anterior cingulate cortex (ACC). mAChRs are known to play crucial roles in cognition, memory, learning, as well as mental illness such as depression and schizophrenia. It is reported that mAChRs regulates excitatory and inhibitory synaptic transmission of SST and PV interneurons in ACC in a cell-type-specific manner. These findings suggest that the antidepressant effects of scopolamine may be attributed to its modulation of synaptic activity within these specific interneuron populations.

Yang et al. have examined the effect of transcranial direct current stimulation (tDCS) on the rehabilitation of cognitive impairment in mild to moderate post-stroke patients. Patients underwent 14 tDCS sessions that lasts 2-weeks, and their cognitive function and functional near-infrared spectroscopy (fNIRS) data of baseline and follow-up were collected. This study reported that the cognitive and brain functions of patients were impaired at baseline, but recovered to a certain extent after tDCS treatment. It also suggested that the increase of cortical activation and functional connectivity between the bilateral cerebral hemispheres measured by fNIRS can be used as biomarkers to evaluate the effectiveness of tDCS treatment in stroke.

The captivating articles within this Research Topic explored the impact of transcranial direct current stimulation (tDCS) on cognitive rehabilitation in mild to moderate post-stroke patients The findings indicated the effectiveness of tDCS, highlighting the increased cortical activation and enhanced functional connectivity between the bilateral cerebral hemispheres as promising biomarkers for assessing the efficacy of tDCS in stroke rehabilitation. Other articles also reflect the diversity and depth into knowing the cognition in psychiatry disorders or neurology disorders. We anticipate that academics and clinicians can derive significant benefits from this Research Topic, gaining insights into the neural mechanisms underlying cognitive deficits and informing their choice of treatment therapies.

## Author contributions

The author confirms being the sole contributor of this work and has approved it for publication.

